# TyG-Centered Endocrine–Metabolic Architecture in Patients with Thyroid Dysfunction: A Systems Phenotyping Study

**DOI:** 10.3390/metabo16070490

**Published:** 2026-07-12

**Authors:** Mirela Frandes, Adriana Gherbon, Anca Tudor, Oana Albai, Mihaela Maria Vlad, Călin Muntean

**Affiliations:** 1 Department of Functional Sciences—Medical Informatics and Biostatistics, “Victor Babes” University of Medicine and Pharmacy, 300041 Timisoara, Romania; mirela.frandes@umft.ro (M.F.); cmuntean@umft.ro (C.M.); 2 Center for Modeling Biological Systems and Data Analysis, “Victor Babes” University of Medicine and Pharmacy, 300041 Timisoara, Romania; 3 Department VII Internal Medicine—Diabetes, Nutrition, Metabolic Diseases and Systemic Rheumatology, “Victor Babes” University of Medicine and Pharmacy, 300041 Timisoara, Romania; albai.oana@umft.ro; 4 Diabetes, Nutrition, and Metabolic Diseases, “Pius Brînzeu” Emergency Hospital, 300723 Timisoara, Romania; 5 Center of Molecular Research in Nephrology and Vascular Disease, “Victor Babes” University of Medicine and Pharmacy, 300041 Timisoara, Romania; vlad.mihaela@umft.ro; 6 Department of Internal Medicine II, University Clinic of Endocrinology, “Victor Babeș” University of Medicine and Pharmacy Timișoara, 300041 Timisoara, Romania; 7 Department of Endocrinology, “Pius Brînzeu” County Emergency Clinic Hospital Timișoara, 300723 Timisoara, Romania

**Keywords:** triglyceride–glucose index, insulin resistance, metabolic syndrome, systems phenotyping, unsupervised clustering, latent profile analysis, Gaussian graphical model, thyroid dysfunction, thyroid autoimmunity

## Abstract

Background: Metabolic dysfunction is a complex process arising from coordinated interactions among insulin resistance, dyslipidemia, hepatic dysfunction, obesity, low-grade inflammation, and endocrine alterations. The triglyceride–glucose (TyG) index is a simple, reproducible surrogate of insulin resistance, but most studies have evaluated it through isolated association-based analyses. We characterized the endocrine–metabolic architecture associated with TyG in patients with thyroid dysfunction using a systems-phenotyping approach. Methods: In this retrospective, single-center, cross-sectional study of 387 adults evaluated for thyroid dysfunction, unsupervised phenotypes were derived by principal component analysis (PCA), K-means, Gaussian mixture models (GMM), and hierarchical (Ward) clustering using fully measured primary metabolic variables (fasting glucose, triglycerides, HDL- and LDL-cholesterol, body mass index, alanine, and aspartate aminotransferase). TyG was then examined as a descriptor of the identified architecture. Model-based latent profile analysis (BIC, entropy) and a censoring-aware, rank-based Gaussian graphical model (GGM) of partial correlations were additionally estimated. Results: Three overlapping, clinically interpretable phenotypic partitions of an underlying metabolic continuum were identified: a metabolically preserved phenotype (MP), an intermediate hepatic-dominant phenotype (IHD), and a severe insulin-resistance-dominant phenotype (SIRD), characterized by obesity, atherogenic dyslipidemia, and hyperuricemia. TyG values increased monotonically across the three partitions (medians 8.24, 8.74, and 8.87 in MP, IHD, and SIRD, respectively; *p* < 0.001), providing an accessible single-number summary of the metabolic gradient. Cluster separation was modest (silhouette = 0.17), consistent with a continuum, but the three-profile solution was supported by latent profile analysis (ΔBIC = −96.7; relative entropy = 0.75) and was reproduced across frameworks (adjusted Rand index of 0.75 with GMM and 0.46 with Ward). In the censoring-aware GGM, metabolic and thyroid variables formed two near-orthogonal blocks (maximum absolute metabolic–thyroid partial correlation: 0.16; median: 0.05). Conclusions: The systems phenotyping approach identified three clinically interpretable phenotypic partitions (MP, IHD, SIRD) along a metabolic continuum in patients evaluated for thyroid dysfunction. TyG values increased monotonically across partitions (medians 8.24, 8.74, 8.87) and provided a pragmatic single-number summary of the principal metabolic axis. The IHD phenotype indicates that hepatic dysregulation can emerge as a distinct early stage rather than solely a late consequence of insulin resistance. Metabolic and thyroid axes were near-orthogonal, positioning thyroid autoimmunity as a parallel modulatory layer. These findings support TyG as an accessible descriptor for multidimensional endocrine–metabolic risk stratification.

## 1. Introduction

Metabolic dysfunction is a complex process involving coordinated interactions among insulin resistance, dyslipidemia, obesity, hepatic dysfunction, chronic low-grade inflammation, and endocrine alterations. Growing evidence suggests that these abnormalities do not occur in isolation, but rather as interconnected biological networks associated with heterogeneous metabolic risk profiles and differential clinical trajectories [1,2]. The triglyceride–glucose (TyG) index has emerged as a simple, reproducible biomarker of insulin resistance derived from fasting triglyceride and glucose concentrations [3,4,5], with growing evidence supporting its value as an integrative indicator of metabolic syndrome, obesity, and type 2 diabetes mellitus (T2DM) [6,7]. Previous studies have associated TyG with metabolic syndrome, T2DM, nonalcoholic fatty liver disease, cardiovascular disease, and hepatic steatosis [8]. However, most previous studies have evaluated TyG using conventional unidimensional analytical frameworks that focus on isolated associations with specific outcomes. Such approaches may incompletely capture the broader endocrine–metabolic organization underlying insulin resistance-related phenotypes. Systems phenotyping strategies based on dimensionality reduction and unsupervised clustering can identify underlying metabolic patterns and biologically coherent endocrine–metabolic phenotypes within heterogeneous patient populations [9]. Patients with thyroid dysfunction represent a particularly relevant model for endocrine–metabolic profiling because they frequently exhibit overlapping metabolic abnormalities, including obesity, dyslipidemia, altered glucose metabolism, hepatic dysfunction, and autoimmune endocrine alterations [10,11]. In this context, TyG may serve as an accessible descriptor of the broader endocrine–metabolic organization identified through systems phenotyping [12]. The aim of the present study was to characterize the endocrine–metabolic organization in patients with thyroid dysfunction and to evaluate how TyG aligns with this organization. Specifically, we aimed to identify data-driven metabolic phenotypes using unsupervised clustering across complementary partitioning, model-based, and hierarchical approaches. We then characterized the partial correlation structure of the endocrine–metabolic system and explored thyroid-autoimmune subphenotypes within the identified metabolic phenotypes.

## 2. Materials and Methods

### 2.1. Study Design and Population

This retrospective cross-sectional study investigated the endocrine–metabolic organization associated with the TyG index in adult patients with thyroid dysfunction. Consecutive adult patients evaluated in the Department of Endocrinology at the “Pius Brînzeu” County Emergency Clinic Hospital, Timișoara, Romania, between 15 March and 15 May 2026, with available metabolic, hepatic, anthropometric, and thyroid-related biochemical data required for TyG calculation and multidimensional endocrine–metabolic profiling, were eligible for inclusion. The study comprised 387 patients with complete endocrine–metabolic datasets available for systems phenotyping. The study was designed and reported in accordance with the STROBE (Strengthening the Reporting of Observational Studies in Epidemiology) guidelines for cross-sectional studies [13]. The study was conducted in accordance with the Declaration of Helsinki and approved by the local institutional ethics committee.

The study evaluated integrated metabolic, hepatic, anthropometric, and endocrine variables, including fasting glucose, triglycerides, HDL cholesterol, LDL cholesterol, body mass index (BMI), alanine aminotransferase (ALT), aspartate aminotransferase (AST), uric acid, thyroid-stimulating hormone (TSH), free thyroxine (FT4), anti-thyroid peroxidase antibodies (antiTPO), and anti-thyroglobulin antibodies (antiTG). Hepatic steatosis was also recorded based on available clinical and imaging evaluations documented in patient records.

All records were pseudonymized prior to analysis: direct identifiers were removed and replaced with a sequential numeric patient identifier, and no personally identifiable information was used in or distributed with any analytic file. Of the eligible patients, all were retained for the primary clustering analysis, which used seven continuous metabolic variables with complete data. No patient was excluded by listwise deletion at this stage, so the three derived phenotypes together account for all patients.

Before analysis, the dataset was audited for data integrity artifacts. Five biochemical variables exhibited pronounced point masses exactly at the cohort median, a pattern consistent with prior constant (median) imputation of missing values rather than with a biological distribution: uric acid (130/387 cells at 5.1 mg/dL; measured *n* = 257), antiTPO (133 cells at 39.65 IU/mL; measured *n* = 254), antiTG (215 cells at 1.3 IU/mL; measured *n* = 172), TSH (23 cells; measured *n* = 364), and FT4 (25 cells; measured *n* = 362). These artefactual point masses were reverted to missing values before any descriptive or inferential analysis, and all tables and analyses now use only measured data for the affected variables, with the denominator explicitly stated. The thyroid autoantibodies were also interval-censored at assay limits of detection: antiTPO was left-censored at 28 IU/mL (90 values) and right-censored/capped at 1300 IU/mL (38 values), and anti-TG showed a similar pattern. These variables were therefore not treated as fully continuous biological measurements. For network estimation, they were rank-transformed (normal scores), an approach equivalent to standard limit-of-detection substitution [14].

### 2.2. Clinical and Biochemical Variables

Anthropometric, metabolic, hepatic, and endocrine-related biochemical variables were collected from the available clinical records and laboratory databases. Continuous variables included fasting glucose, triglycerides, HDL cholesterol, LDL cholesterol, BMI, ALT, AST, uric acid, TSH, FT4, antiTPO, and antiTG values.

The TyG index was calculated as [4,5]:

$$TyG=ln\left(\frac{Triglycerides\times Glucose}{2}\right)$$

where fasting triglyceride and fasting glucose concentrations were expressed in mg/dL.

Obesity was defined as BMI ≥ 30 kg/m^2^. Hyperuricemia was defined as uric acid > 6 mg/dL. Hypothyroidism was defined as a TSH > 4.5 mIU/L. AntiTPO positivity was defined using antiTPO values > 34 IU/mL, and antiTG positivity using antiTG values > 4.5 IU/mL, consistent with the local laboratory reference ranges. An autoimmune thyroiditis proxy variable was additionally constructed as positivity for antiTPO and/or antiTG, with priority given to antiTPO when both assays were available. Hepatic steatosis was defined as a binary variable, diagnosed by abdominal ultrasound, and documented in patient records.

Thyroid functional status was interpreted in the context of treatment rather than based solely on TSH. In this tertiary-referral cohort, 251 of 387 patients (64.9%) were receiving levothyroxine substitution, and 120 (31.0%) had undergone thyroid surgery or radioiodine therapy; only 4 untreated patients had TSH > 4.5 mIU/L, so frank biochemical hypothyroidism was rare.

### 2.3. Statistical Analysis

Continuous variables were assessed for distributional characteristics and are presented as medians and interquartile ranges (IQRs). Categorical variables are presented as counts and percentages. Comparisons across TyG quartiles and identified endocrine–metabolic phenotypes were performed using the Kruskal–Wallis H test for continuous variables and the chi-square test for categorical variables. Pairwise post hoc comparisons used Dunn’s test for continuous variables and Fisher’s exact test for categorical variables, with the Holm correction for multiple comparisons [15]. The reported pairwise *p*-values are Holm-adjusted. Within-phenotype thyroid-oriented sub-clustering comparisons were evaluated using the Mann–Whitney U test.

Continuous inputs were preprocessed using an explicit, reproducible pipeline: (i) mild outlier truncation via winsorization at the 1st and 99th percentiles; (ii) natural-log transformation of right-skewed variables (|skewness| > 1: fasting glucose, triglycerides, ALT, AST); and (iii) z-score standardization. All analyses were performed using R statistical software, version 4.5.1 (R Foundation for Statistical Computing, Vienna, Austria). K-means clustering, Ward’s hierarchical clustering, Gaussian mixture modeling, latent profile analysis with Bayesian information criterion and entropy, the silhouette coefficient, and the Adjusted Rand Index (ARI) were implemented using the stats, cluster, and mclust packages. The Gaussian graphical model was estimated with the huge package using a nonparanormal (rank-based) transformation, followed by graphical lasso regularization with cross-validated penalty selection (optimal α = 0.068). Network visualization used qgraph, whereas data handling and figures used the tidyverse, ggplot2, and ComplexHeatmap packages. A model-based latent profile analysis was also estimated to provide an information-criterion- and entropy-based assessment of cluster solidity.

### 2.4. Multidimensional Endocrine–Metabolic Profiling

An endocrine–metabolic profiling strategy was used to investigate the underlying metabolic organization associated with the TyG index in the study cohort. Continuous metabolic, hepatic, anthropometric, and endocrine variables were standardized before analysis. Variables with skewed distributions underwent logarithmic transformation before standardization, and mild outliers were also truncated to reduce the influence of extreme outliers [16].

Principal component analysis (PCA) was applied to standardized primary metabolic variables to identify the dominant axes of endocrine–metabolic variability in the cohort. PCA loadings were analyzed to characterize coordinated relationships among glycemic, lipid, hepatic, and anthropometric parameters, and the alignment of TyG with the principal axis was quantified using Spearman’s correlation between TyG and PC1. The resulting low-dimensional structure was further examined to identify biologically coherent endocrine–metabolic phenotypes.

### 2.5. Identification of Metabolic Phenotypes

Because TyG is, by construction, a deterministic log-linear function of fasting triglycerides and glucose, the unsupervised analyses (PCA, K-means, Gaussian mixture model, hierarchical clustering, Gaussian graphical model, and latent profile analysis) were run on the seven primary metabolic variables (fasting glucose, triglycerides, HDL-cholesterol, LDL-cholesterol, BMI, ALT, AST) to obtain a data-driven metabolic continuum independent of TyG. Both TyG and uric acid were then examined as descriptors of the identified phenotype structure: TyG quartiles, the median TyG per independently identified phenotype, and the Spearman correlation of TyG with PC1 were used to quantify the alignment between TyG and the principal metabolic axis. Throughout the manuscript, the seven primary metabolic variables (fasting glucose, triglycerides, HDL-cholesterol, LDL-cholesterol, BMI, ALT, AST) are designated as cluster-defining variables; TyG, uric acid, and the thyroid-related variables (TSH, FT4, anti-TPO, anti-TG) are designated as descriptive variables used for post hoc characterization of the data-driven phenotypes and are not used in cluster derivation.

The conditional dependence structure of the endocrine–metabolic system was estimated using a Gaussian graphical model (GGM). To account for the non-normal, censored distributions of the antibodies, the GGM was estimated on rank-transformed (normal-score) variables, a nonparanormal specification [17], with a regularized (graphical-lasso) precision matrix. The regularization parameter was selected by cross-validation (α = 0.068) [18]. Consistent with its role as a descriptor of the identified architecture, TyG was examined separately rather than as a network node. Partial correlations were inspected after conditioning on all other variables, and metabolic–thyroid edges were summarized by their maximum and median absolute values.

**Figure 1:**
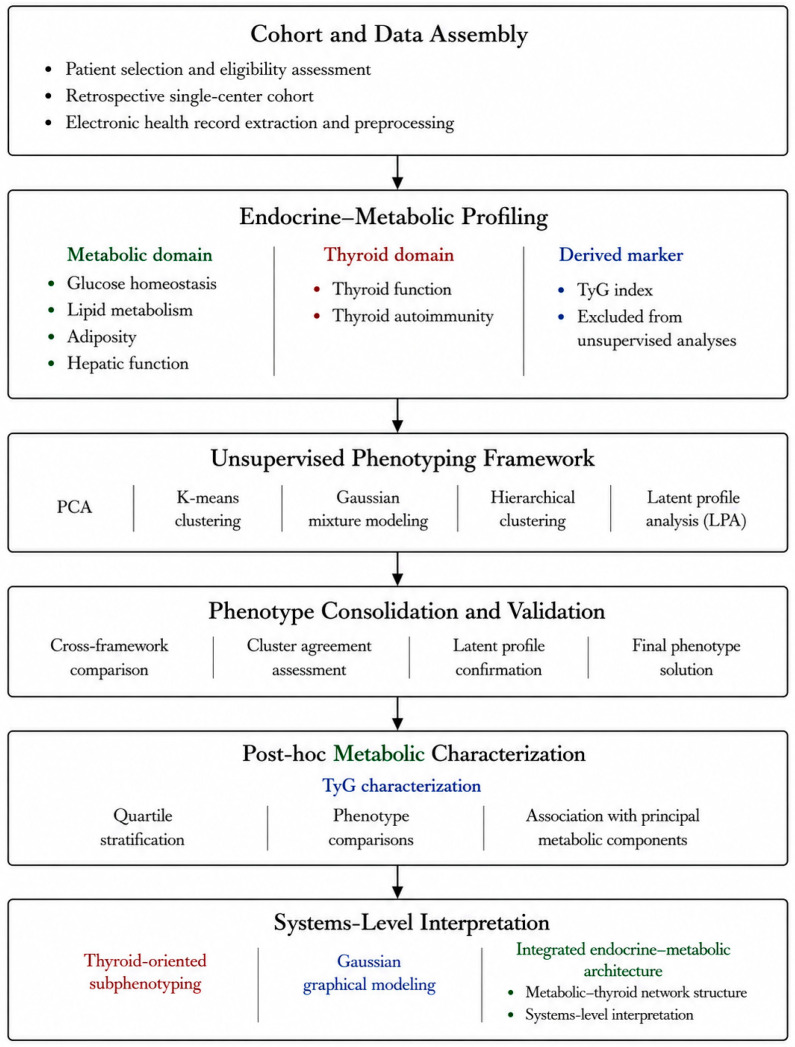
Workflow of the multidimensional endocrine–metabolic systems phenotyping approach. Abbreviations: TyG, triglyceride–glucose index; PCA, principal component analysis; GMM, Gaussian mixture model; ARI, Adjusted Rand Index.

After phenotype identification, phenotype-specific metabolic profiles were characterized by comparing glycemic, lipid, hepatic, anthropometric, and endocrine variables across clusters. Secondary within-phenotype clustering analyses were also performed using thyroid-related variables, including TSH, FT4, and thyroid autoantibodies, to explore thyroid-autoimmune substructure within the identified metabolic phenotypes. The overall analytical workflow for multidimensional endocrine–metabolic systems phenotyping is illustrated in Figure 1 and includes data preprocessing, dimensionality reduction, clustering analyses, phenotype identification, and exploration of thyroid-autoimmune substructure.

The analytical pipeline integrates five sequential stages (Figure 1): (i) data assembly and preprocessing of the study cohort, including computation of the TyG index, logarithmic transformation of skewed laboratory variables, mild outlier truncation, and z-score standardization across seven primary metabolic clustering variables (fasting glucose, triglycerides, BMI, ALT, AST, LDL, HDL); (ii) dimensionality reduction via PCA to identify the dominant axes of endocrine–metabolic variability, characterize their loadings, and assess the alignment of TyG with the principal metabolic axis; (iii) unsupervised phenotype identification using K-means clustering with silhouette-based selection of the optimal number of clusters; (iv) robustness verification of the K-means solution via hierarchical agglomerative (Ward’s method) and GMM clustering, with cross-method concordance quantified by the ARI; and (v) secondary thyroid-oriented sub-clustering analyses within each major endocrine–metabolic phenotype to explore additional thyroid-autoimmune substructure.

## 3. Results

### 3.1. Baseline Characteristics of the Study Cohort

Baseline metabolic and endocrine characteristics showed wide distributions across the cohort, with broad variability in TyG, hepatic enzymes, lipid parameters, BMI, and thyroid markers (Table 1). Across increasing TyG quartiles, the cohort exhibited a coordinated metabolic gradient with progressive elevations in fasting glucose, triglycerides, BMI, uric acid, and hepatic transaminases, together with a corresponding decrease in HDL cholesterol (Kruskal–Wallis H tests, *p* < 0.001). In contrast, thyroid-related variables (TSH, FT4, antiTPO, antiTG) remained relatively stable across quartiles.

### 3.2. Endocrine–Metabolic Organization and TyG Gradient

To further explore the multidimensional endocrine–metabolic organization of the study cohort, patients were stratified according to TyG index quartiles. Progressive changes across TyG quartiles revealed a structured metabolic gradient characterized by coordinated alterations in glycemic, lipidic, hepatic, anthropometric, and endocrine-related parameters. Higher TyG quartiles were associated with progressively higher fasting glucose, triglyceride levels, BMI, hepatic enzymes, and uric acid levels, supporting the integration of TyG into a broader insulin resistance-associated metabolic axis. In contrast, HDL cholesterol demonstrated an inverse trend across increasing TyG categories, consistent with the progressive emergence of an adverse metabolic phenotype. The observed quartile-dependent metabolic organization further suggested coordinated hepato-metabolic remodeling, as reflected in rising ALT and AST levels across higher TyG categories. These findings support the concept that TyG reflects an integrated endocrine–metabolic framework linking dysglycemia, lipid dysregulation, adiposity, and hepatic metabolic stress.

Thyroid-related variables showed a distinct distribution across TyG quartiles. Although thyroid-associated parameters contributed to the cohort’s overall endocrine–metabolic heterogeneity, their progression across TyG categories was less linear and less dominant than that of classical metabolic variables. This pattern suggested that thyroid-related alterations may act as modulatory endocrine components operating alongside the predominantly metabolic–hepatic organizational structure. The progressive endocrine–metabolic reorganization observed across TyG quartiles provided the rationale for subsequent unsupervised clustering analyses to identify endocrine–metabolic phenotypes within the cohort.

**Figure 2:**
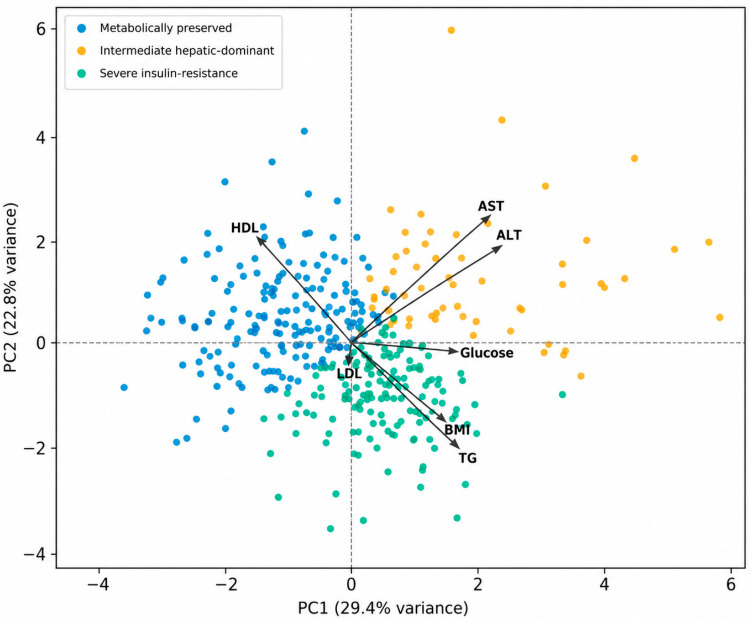
Principal component analysis (PCA) of the standardized primary metabolic variables. The first three components account for 67.5% of the total variance (PC1, 29.4%; PC2, 22.8%; PC3, 15.3%). PC1 represents a combined insulin-resistance–hepatic axis; PC2 reflects a hepatic–dyslipidemic dimension. Abbreviations: PCA, principal component analysis; PC, principal component; TyG, triglyceride–glucose index; HDL, high-density lipoprotein; LDL, low-density lipoprotein; BMI, body mass index; ALT, alanine aminotransferase; AST, aspartate aminotransferase.

PCA of the standardized primary metabolic variables revealed a structured, low-dimensional organization. The first two principal components accounted for 52.2% of the cumulative variance (PC1, 29.4%; PC2, 22.8%), with PC3 contributing an additional 15.3% (cumulative 67.5%). PC1 loadings were dominated by ALT (+0.55) and AST (+0.50), with substantial contributions from fasting glucose (+0.43), triglycerides (+0.35), BMI (+0.29), and HDL (−0.26), consistent with a coordinated insulin-resistance–hepatic axis. PC2 was defined by an HDL (+0.50) versus triglycerides (−0.48) contrast, together with hepatic transaminases (ALT +0.38, AST +0.47), reflecting a partially separable hepatic–dyslipidemic dimension of variability (Figure 2). In a post hoc analysis, TyG correlated with PC1 (Spearman’s ρ = 0.69); however, because triglycerides and glucose, the mathematical components of TyG, were among the clustering inputs, this correlation is partly expected by construction. A sensitivity PCA restricted to the five non-component metabolic variables (HDL, LDL, BMI, ALT, AST) yielded a hepatic-dominated axis on which TyG showed only a weak residual correlation (ρ = 0.15), consistent with TyG primarily reflecting the lipid–glucose–adiposity dimension of the continuum rather than the hepatic axis.

**Figure 3:**
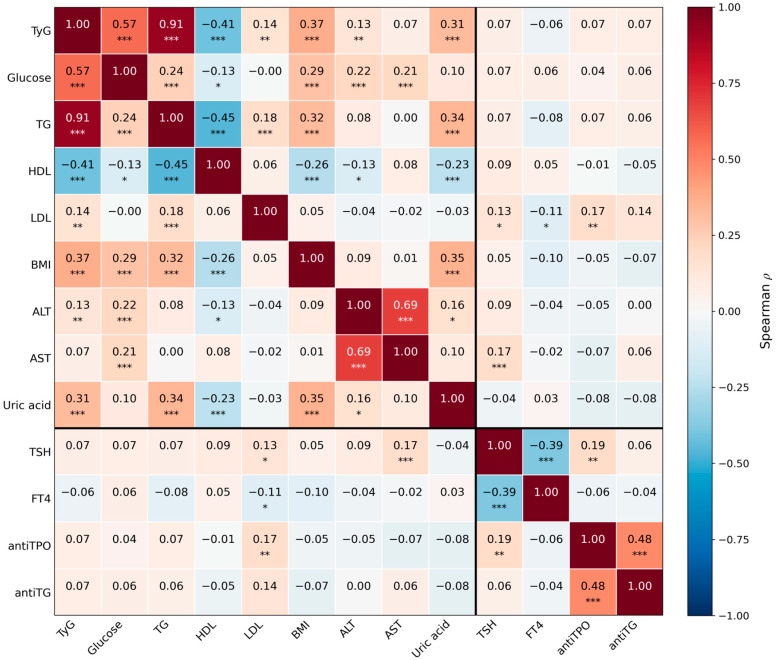
Correlation heatmap of endocrine–metabolic and thyroid-related variables in the study cohort. Pairwise Spearman correlation coefficients are displayed for the endocrine–metabolic variables used for primary clustering and, in a separately demarcated block, the thyroid-related variables. Each cell reports ρ; asterisks denote statistical significance: * *p* < 0.05; ** *p* < 0.01; *** *p* < 0.001. Abbreviations: TyG, triglyceride–glucose index; ALT, alanine aminotransferase; AST, aspartate aminotransferase; HDL, high-density lipoprotein; LDL, low-density lipoprotein; BMI, body mass index; TSH, thyroid-stimulating hormone; FT4, free thyroxine; antiTPO, anti-thyroid peroxidase antibodies; antiTG, anti-thyroglobulin antibodies.

The correlation structure of the endocrine–metabolic and thyroid-related variables is illustrated in Figure 3, which demonstrates a coordinated metabolic axis linking TyG to glycemic, lipidic, hepatic, and anthropometric parameters, whereas thyroid-related variables exhibit comparatively weaker and more heterogeneous associations.

### 3.3. Identification of Data-Driven Endocrine–Metabolic Phenotypes

Unsupervised clustering analysis identified three overlapping yet clinically interpretable endocrine–metabolic phenotypes in the study cohort, revealing progressive patterns of endocrine and metabolic organization beyond conventional linear TyG stratification. The selected three-cluster solution demonstrated metabolic coherence and clinical interpretability, supporting its use as a parsimonious partition of a continuous endocrine–metabolic gradient rather than as a representation of sharply distinct biological entities.

**Figure 4:**
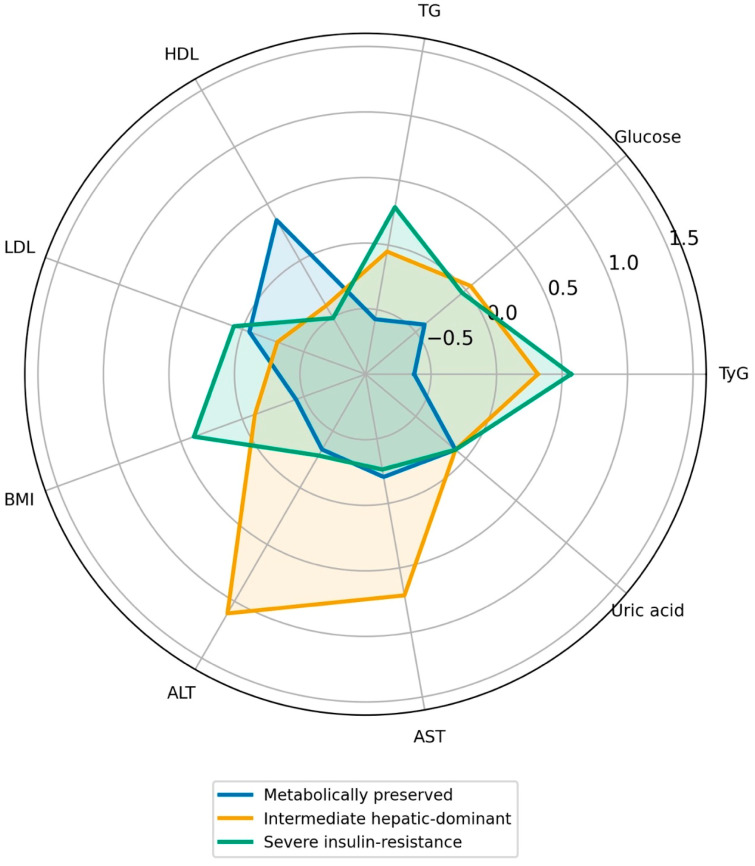
Data-driven endocrine–metabolic phenotypes identified by unsupervised K-means clustering. Radar plots illustrate the relative profiles of the three identified endocrine–metabolic phenotypes. Abbreviations: TyG, triglyceride–glucose index; HDL, high-density lipoprotein; LDL, low-density lipoprotein; BMI, body mass index; ALT, alanine aminotransferase; AST, aspartate aminotransferase; TSH, thyroid-stimulating hormone; FT4, free thyroxine; antiTPO, anti-thyroid peroxidase antibodies; antiTG, anti-thyroglobulin antibodies.

The identified clusters differed substantially across glycemic, lipidic, hepatic, anthropometric, and thyroid-related dimensions, indicating heterogeneous endocrine–metabolic remodeling patterns associated with insulin resistance. The overall cluster structure is illustrated in Figure 4, and the detailed characteristics of each cluster are summarized in Table 2.

The metabolically preserved (MP) cluster was characterized predominantly by a relatively favorable metabolic profile, with lower TyG values, lower triglyceride concentrations, lower hepatic enzyme levels, and comparatively preserved HDL cholesterol levels. This phenotype had the lowest overall metabolic burden within the cohort and was associated with comparatively limited hepatic and adiposity-related metabolic disturbance.

The intermediate hepatic-dominant (IHD) cluster was characterized by elevated hepatic transaminases (ALT, AST), higher BMI, more frequent hepatic steatosis, hyperuricemia, and a higher prevalence of hypothyroidism. The IHD represents a transitional metabolic phenotype situated between relatively preserved metabolic organization and advanced insulin resistance-associated dysfunction, in which hepatic dysregulation emerges as an early and partially independent component of the metabolic continuum.

The severe insulin-resistance-dominant (SIRD) cluster exhibited the most pronounced insulin-resistance-associated metabolic remodeling profile, characterized by elevated TyG values, hypertriglyceridemia, elevated fasting glucose, elevated hepatic enzyme levels, elevated uric acid levels, and adverse lipid remodeling. This phenotype represented the highest endocrine–metabolic burden within the cohort and showed a coordinated pattern of insulin resistance-associated hepatic–metabolic dysfunction. Although the principal organizational structure of the identified clusters remained predominantly hepatic-metabolic, thyroid-related variables exhibited heterogeneous distributions across clusters, suggesting the presence of additional endocrine modulatory substructures not fully captured by the primary clustering solution. These observations provided the rationale for subsequent thyroid-oriented subclustering analyses within the identified metabolic phenotypes.

Unsupervised clustering identified three distinct data-driven endocrine–metabolic phenotypes, characterized by MP, IHD, and SIRD, as illustrated in Figure 4. Differences in clinical and endocrine characteristics across the phenotypes were assessed using the Kruskal–Wallis H test for continuous variables and the chi-square test with pairwise post hoc comparisons for categorical variables (Table 2). TyG values increased monotonically across the three partitions (median 8.24, 8.74, and 8.87 in MP, IHD, and SIRD), providing an accessible single-number summary of the metabolic gradient identified by the unsupervised analysis. Post hoc analyses showed that SIRD had a significantly higher prevalence of obesity than MP (post hoc *p* < 0.001). Similarly, hyperuricemia was significantly more frequent in SIRD than in MP (post hoc *p* < 0.001), supporting a markedly increased metabolic burden in this subgroup. The IHD showed a partially overlapping metabolic profile that fell between MP and SIRD, consistent with a transitional endocrine–metabolic state characterized predominantly by hepatic involvement.

Thyroid-related alterations also showed heterogeneous distributions across the identified phenotypes. Hypothyroidism prevalence differed significantly between IHD and MP (post hoc, *p* = 0.004) and between IHD and SIRD (post hoc, *p* = 0.010). In contrast, thyroid autoantibody positivity showed less pronounced separation between clusters. Overall, these findings support the concept that the systems phenotyping approach captures clinically meaningful endocrine–metabolic heterogeneity that extends beyond isolated biochemical variation. Although the primary clustering structure was driven by metabolic and hepatic variables, heterogeneous thyroid-related distributions within clusters suggested the presence of additional thyroid-oriented substructures, prompting subsequent sub-clustering analyses within each identified phenotype.

### 3.4. Robustness Across Clustering Frameworks

**Figure 5:**
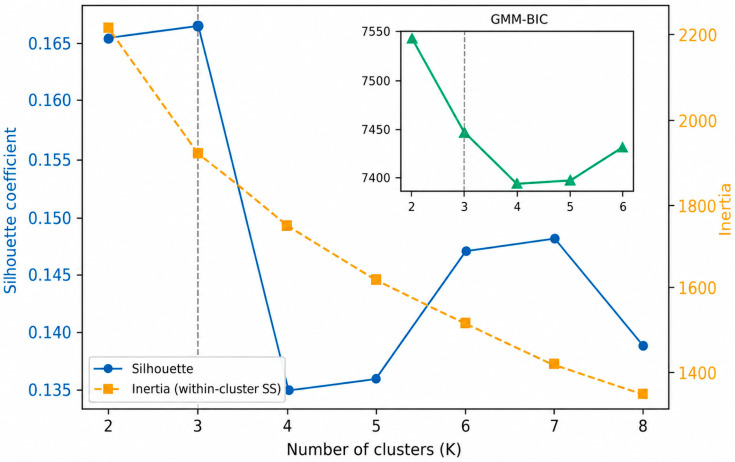
Cluster validation across candidate numbers of clusters (K = 2 to 8). Silhouette coefficient (circles, left *y*-axis) and inertia (within-cluster sum of squares; squares, right *y*-axis) are shown for each candidate K.

Alternative clustering approaches, including hierarchical agglomerative clustering (Ward’s method) and Gaussian mixture modeling (diagonal covariance), reproduced the principal TyG-associated endocrine–metabolic organization identified by the primary K-means framework. Cluster separation was modest in absolute terms (silhouette coefficient 0.166 for K = 3, marginally exceeding 0.165 for K = 2 and higher than 0.135–0.148 for K = 4–8), consistent with metabolic dysregulation forming a continuum rather than sharply separated clusters (Figure 5). Quantitative cross-method concordance, assessed using the Adjusted Rand Index (ARI), was substantial between K-means and GMM (ARI = 0.75) and moderate between K-means and Ward (ARI = 0.46). The three-profile solution was further supported by latent profile analysis (ΔBIC = −96.7 at K = 3 versus K = 2; relative entropy = 0.75), indicating reasonable but not categorical class separability, again consistent with a continuum. Notably, the IHD was consistently identified across all three frameworks, indicating that this stratum is not an artifact of the K-means partition. Overall, the preservation of the principal endocrine–metabolic structure across distinct clustering strategies supports the relative stability and biological coherence of the identified phenotypes.

### 3.5. Sensitivity Analyses

To address potential confounding by treatment status and by sex composition, two sensitivity analyses were performed. In a treatment-naïve subgroup (*n* = 117 patients with no current levothyroxine substitution and no prior thyroid surgery or radioiodine therapy), the three-phenotype partition was preserved, with cluster proportions comparable to the overall cohort (MP 53.8%, IHD 12.8%, SIRD 33.3%) and an identical monotonic TyG gradient across phenotypes (medians 8.25, 8.81, and 8.93 in MP, IHD, and SIRD, respectively). The IHD hepatic signature remained equally pronounced (median ALT 51 vs. 20 and 21 U/L; median AST 38 vs. 21 and 21 U/L in IHD vs. MP and SIRD). In the nonparanormal partial-correlation network restricted to this subgroup, metabolic–thyroid edges remained weak in absolute terms but were slightly less attenuated than in the full cohort (maximum absolute partial *r* = 0.29; median = 0.08), consistent with near-orthogonality of the two axes while suggesting that treatment status may partially modulate the magnitude of cross-block correlations.

Because the IHD cluster contained a higher proportion of male patients than the other phenotypes (38.6% vs. 15.7% in MP and 20.0% in SIRD), and hepatic transaminases may differ by sex, the IHD hepatic signature was tested separately by sex. The signature was robustly present in both sexes: median ALT was 46 vs. 20 U/L in IHD vs. non-IHD women (Mann–Whitney U test, *p* < 0.001) and 50.5 vs. 22.5 U/L in IHD vs. non-IHD men (*p* < 0.001); analogous results were obtained for AST (women: 36 vs. 21 U/L, *p* < 0.001; men: 38 vs. 22 U/L, *p* < 0.001). The IHD hepatic phenotype is therefore not an artifact of sex composition.

### 3.6. Thyroid-Autoimmune Substructure Within Metabolic Phenotypes

Although the principal clustering structure was predominantly driven by metabolic and hepatic variables, substantial heterogeneity in thyroid-related parameters remained observable within each identified phenotype. To further describe this endocrine heterogeneity, secondary thyroid-oriented subclustering analyses were performed within each major data-driven phenotype (Table 3). Because these subclusters were defined using thyroid-related variables (TSH, FT4, antiTPO, antiTG) and subsequently characterized with the same variables, the analyses are descriptive and do not constitute independent validation of distinct thyroid sub-phenotypes.

Within MP, two thyroid-associated sub-phenotypes were identified among patients with available autoantibody data (Table 3): a low thyroid-autoimmune subgroup and a dominant thyroid-autoimmune subgroup. The thyroid-autoimmune dominant subgroup showed markedly higher antiTPO and antiTG levels than the thyroid-autoimmune low subgroup while maintaining a relatively preserved metabolic profile. As expected from the subclustering definition, the autoimmune-dominant subgroup showed significantly higher autoantibody levels, illustrating autoimmune heterogeneity even among patients with comparatively preserved metabolic organization.

Within IHD, thyroid-oriented sub-clustering described additional endocrine heterogeneity, characterized by variable thyroid autoimmunity burden and thyroid functional alterations. The thyroid-autoimmune-dominant subgroup exhibited higher thyroid autoantibody levels and relative alterations in thyroid functional markers, suggesting a more pronounced endocrine modulation alongside the hepatic-metabolic phenotype. Similarly, within SIRD, secondary sub-clustering identified analogous autoantibody-based sub-phenotypes. The thyroid-autoimmune-dominant subgroup demonstrated significantly higher antiTPO and antiTG levels than the thyroid-autoimmune-low subgroup, while severe metabolic dysregulation was preserved in both sub-phenotypes. These findings suggest that thyroid autoimmunity may represent an additional endocrine layer interacting with severe insulin-resistance states rather than serving as a primary determinant of the principal metabolic phenotype. Overall, these findings support the concept that data-driven endocrine–metabolic phenotypes contain thyroid-associated substructures, reflecting a broader endocrine organization that extends beyond isolated metabolic dysregulation.

### 3.7. Model-Based Confirmation and Partial-Correlation Network Architecture

**Figure 6:**
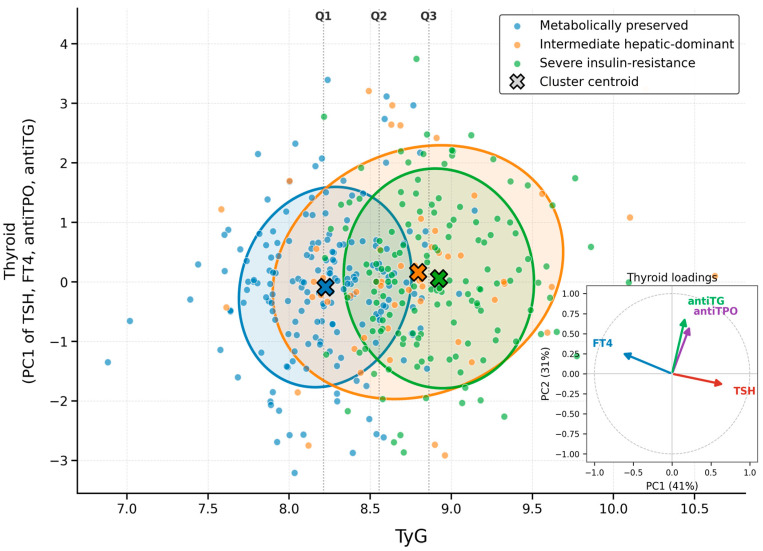
Endocrine–metabolic architecture in a TyG-anchored two-axis projection. Points are color-coded by K-means metabolic phenotype (blue: metabolically preserved [MP]; orange: intermediate hepatic-dominant [IHD]; green: severe insulin-resistance-dominant [SIRD]); filled ellipses depict 1.5-σ cluster densities, and X markers indicate cluster centroids. Dotted vertical lines mark TyG quartile boundaries (Q1, Q2, Q3). Inset: loadings of the four thyroid variables on PC1 × PC2, with arrows colored by variable (blue: FT4; red: TSH; green: antiTG; purple: antiTPO). Abbreviations: TyG, triglyceride–glucose index; PC1, first principal component; TSH, thyroid-stimulating hormone; FT4, free thyroxine; antiTPO, anti-thyroid peroxidase antibodies; antiTG, anti-thyroglobulin antibodies.

The overall endocrine–metabolic organization is summarized in Figure 6, where all patients are projected onto two principal axes: the TyG index and the first principal component of the four thyroid variables. The three metabolic phenotypes separate predominantly along the TyG axis, recapitulating the primary clustering, while their distributions overlap almost entirely along the thyroid composite axis, a visual illustration that the TyG-aligned metabolic gradient and the thyroid axis are quantitatively orthogonal dimensions. This block-diagonal organization complements the within-cluster thyroid-autoimmune stratification documented above, indicating that the metabolic and thyroid layers form two largely independent dimensions of the endocrine–metabolic system.

To provide a formal, model-based check of the parsimony of the three-cluster solution, latent profile analysis (Gaussian mixture model with diagonal covariance, K = 2 to K = 6) was conducted on the same seven standardized primary metabolic variables. The Bayesian Information Criterion (BIC) showed a clear improvement from K = 2 to K = 3 (ΔBIC = −96.7), with smaller incremental improvements thereafter, supporting the three-cluster solution as a parsimonious and statistically supported representation. The relative entropy of classification under K = 3 was 0.75, indicating reasonable but not categorical class separability, again consistent with the continuum interpretation of the metabolic gradient. Together with the modest silhouette coefficient (0.17) and the substantial-to-moderate cross-method concordance (ARI 0.75 with GMM and 0.46 with Ward), these results support K = 3 as a clinically interpretable and statistically defensible partition of the underlying endocrine–metabolic continuum.

The conditional-dependence structure was estimated using a censoring-aware, rank-based (nonparanormal) Gaussian graphical model with a cross-validated graphical lasso penalty (α = 0.068), excluding TyG. The metabolic and hepatic variables formed a densely connected block (ALT–AST partial *r* = 0.66; TG–HDL negative), whereas thyroid variables formed a compact separate substructure (TSH–FT4 negative; antiTPO–antiTG positive). Crucially, all metabolic–thyroid partial correlations were uniformly weak (maximum |partial *r*| = 0.16; median = 0.05), indicating that the metabolic and thyroid axes operate as conditionally near-independent dimensions of the endocrine–metabolic system rather than as components of a single integrated structure. This represents a substantial methodological refinement over Pearson-based GGM estimates that are sensitive to non-normality and to the censored, point-mass distributions of the autoantibodies. The block-diagonal nature of this conditional-dependence architecture is mirrored by the orthogonal organization of patients in the two-axis projection in Figure 6.

## 4. Discussion

Using a systems phenotyping approach in which TyG was used as a descriptor of the data-driven metabolic continuum and of the independently identified phenotypes, we identified three overlapping, clinically interpretable phenotypic partitions of the endocrine–metabolic continuum in patients evaluated for thyroid dysfunction: a metabolically preserved phenotype (MP), an intermediate hepatic-dominant phenotype (IHD), and a severe insulin-resistance-dominant phenotype (SIRD) with obesity, atherogenic dyslipidemia, and hyperuricemia [9]. These phenotypes were reproducible across clustering frameworks and were characterized by distinct patterns of metabolic, hepatic, anthropometric, and endocrine organization. Beyond the principal metabolic structure, secondary thyroid-oriented analyses revealed a near-orthogonal thyroid-autoimmune layer within each metabolic phenotype, indicating endocrine heterogeneity that extends beyond isolated biochemical abnormalities. The TyG values aligned with the phenotypic ordering and provided an accessible clinical surrogate for the broader endocrine–metabolic organization.

The TyG index has become a widely used surrogate marker of insulin resistance because of its simplicity, reproducibility, and accessibility in routine clinical practice [3,4,5,6,7,8]. Previous studies have linked TyG to metabolic syndrome, T2DM, hepatic steatosis, cardiovascular disease, and adverse cardiometabolic outcomes. However, most prior investigations have primarily evaluated TyG using conventional association-based analytical frameworks focused on isolated metabolic outcomes [6,7,8,19]. In contrast, the present study examined TyG within an integrative endocrine–metabolic analytical framework designed to explore underlying patterns of biological organization rather than isolated linear associations. The progressive metabolic reorganization observed across TyG quartiles was characterized by coordinated changes in glycemic control, lipid metabolism, adiposity, hepatic dysfunction, and uric acid metabolism, consistent with the concept that TyG captures integrated metabolic remodeling associated with insulin resistance-related endocrine dysfunction [1,2,7,20].

The unsupervised clustering analyses further extended these observations by identifying biologically coherent endocrine–metabolic phenotypes characterized by differential metabolic burden and hepatic involvement. MP showed comparatively favorable metabolic organization, whereas SIRD showed coordinated dysglycemic, lipidic, hepatic, and uric acid abnormalities consistent with advanced endocrine–metabolic dysfunction [21,22]. Notably, the data-driven analysis did not yield a binary preserved-versus-severe partition; instead, it identified IHD as a distinct stratum within the continuum. This phenotype was characterized by elevated hepatic transaminases, higher BMI, more frequent hepatic steatosis, hyperuricemia, and a higher prevalence of hypothyroidism, indicating that hepatic dysregulation can emerge as an early, partially independent component of the metabolic continuum rather than a late consequence of severe insulin resistance. The reproducibility of this three-phenotype organization across alternative clustering frameworks (K-means, GMM, Ward) supports the robustness of the identified phenotype structure. The position of IHD between the two extremes constitutes one of the most informative pathophysiological observations of the present study.

These findings align with growing evidence that insulin resistance-related disorders are characterized by substantial metabolic heterogeneity rather than a uniform biochemical progression [9,23,24,25]. Previous studies have shown that obesity, hepatic steatosis, dyslipidemia, and glucose dysregulation may organize into partially overlapping metabolic trajectories associated with distinct clinical risk profiles. Within this context, the present results suggest that systems phenotyping may help characterize endocrine–metabolic organization patterns that are incompletely captured by conventional linear metabolic stratification approaches.

Although the principal clustering structure remained predominantly metabolic–hepatic, substantial thyroid-related heterogeneity persisted within clusters [9,10,11,12]. The descriptive thyroid-oriented subclustering identified thyroid-autoimmune-dominant and thyroid-autoimmune-low sub-phenotypes within each metabolic phenotype. Because these sub-clusters were defined using the same thyroid variables used to characterize them, the analyses are presented as descriptive rather than as independent validation. Importantly, these thyroid-oriented substructures did not replace the primary metabolic structure but instead appeared alongside it, suggesting a hierarchical endocrine–metabolic organization. This observation may indicate that thyroid autoimmunity and thyroid functional variability act as modulatory endocrine layers that interact with broader insulin resistance-associated metabolic states, rather than functioning as isolated determinants of metabolic dysfunction [26,27,28,29]. This interpretation is supported by the rank-based GGM, which is resistant to censoring and non-normality in autoantibody distributions. All absolute metabolic–thyroid partial correlations remained ≤0.16 (median = 0.05), positioning thyroid autoimmune burden as a parallel, near-orthogonal dimension of endocrine–metabolic variation in this population. The comparatively weaker and less linear distribution of thyroid-related variables across TyG quartiles further supports this interpretation.

The observed coexistence of metabolic dysregulation and thyroid-autoimmune heterogeneity may reflect complex bidirectional interactions among insulin resistance, adiposity, chronic low-grade inflammation, hepatic dysfunction, and endocrine-immune regulation [30,31,32,33]. Dietary and iatrogenic factors, such as excessive iodine intake from herbal weight-loss supplements, can also modulate thyroid function in patients with metabolic disorders, further underscoring the clinical complexity of the thyroid–metabolic interface [34]. Previous studies have linked thyroid dysfunction to obesity, dyslipidemia, hepatic steatosis, and insulin resistance-related metabolic remodeling. However, the present study extends these observations by suggesting that thyroid-related variability may remain partially obscured within broader endocrine–metabolic organizational structures, as identified through systems phenotyping.

Although the present analyses remain exploratory and cannot directly establish causal mechanisms, the patterns identified are consistent with several well-described pathophysiological pathways. The coordinated co-occurrence of elevated TyG, triglycerides, BMI, hepatic transaminases, and uric acid with reduced HDL cholesterol within SIRD is consistent with the canonical insulin resistance–ectopic fat–lipotoxicity axis, in which adipose tissue dysfunction promotes hepatic lipid accumulation, free fatty acid–driven hepatic insulin resistance, and downstream atherogenic dyslipidemia [11,12,35,36]. The orthogonal thyroid-autoimmune substructure may reflect complementary endocrine–immune mechanisms operating in parallel to the metabolic axis, consistent with reported bidirectional interactions between thyroid function, adiposity, and metabolic inflammation [10,26,27,28,29]. These mechanistic interpretations remain tentative in the absence of molecular and longitudinal data, but the phenotypic structure provides a hypothesis-generating framework for future investigations.

From a clinical perspective, these findings suggest that TyG-aligned metabolic profiling may provide insight into the heterogeneity of endocrine-associated metabolic dysfunction. Rather than a single linear continuum, endocrine–metabolic dysfunction may involve overlapping metabolic and endocrine organizational layers, with variable contributions from the liver, glycemia, lipids, and autoimmunity. Although the present analyses remain exploratory, such profiling strategies may inform future phenotype-oriented approaches to metabolic risk stratification and endocrine–metabolic characterization [9,24]. SIRD captures a metabolic syndrome–like pattern in which coordinated dysglycemia, hypertriglyceridemia, low HDL cholesterol, elevated hepatic enzymes, and hyperuricemia coexist and mutually potentiate one another, providing a phenotypic window into the interconnected pathophysiology of obesity, diabetes, and metabolic syndrome.

The present study has several strengths. The analytical framework integrated endocrine, hepatic, metabolic, and anthropometric variables within a unified systems phenotyping approach. Multiple clustering frameworks were used to assess phenotype robustness, and secondary thyroid-oriented analyses revealed additional endocrine heterogeneity within the identified metabolic phenotypes. Nevertheless, several limitations should be acknowledged. The study had a retrospective cross-sectional design and therefore does not permit causal inference. The analyses were conducted in a single-center cohort, and some subgroup analyses involved relatively small numbers of patients. The study did not include an external control group of obese patients without thyroid dysfunction or of healthy individuals. The metabolic phenotypes and TyG associations identified here therefore characterize the structure of metabolic heterogeneity within a thyroid-dysfunction endocrinology population, rather than benchmarking it against non-endocrine reference groups. External comparison with such control populations remains an important direction for future work. In addition, clustering analyses remain exploratory and may be influenced by cohort composition, variable selection, and preprocessing strategies, although the three-cluster solution was further supported by Bayesian information criterion-based latent profile analysis and by the partial-correlation network. The cohort also reflects a tertiary endocrinology referral population enriched for patients with severe or specialized thyroid pathology (post-thyroidectomy iatrogenic hypothyroidism, Graves’ disease, acromegaly, Cushing’s syndrome, macroadenomas). This limits generalizability to community-based, non-endocrine, or ethnically distinct populations. Furthermore, the presence of previously imputed values and detection-limit-censored laboratory measurements for a subset of biochemical variables may have attenuated within-cluster variability and should be considered when interpreting the magnitude of cluster separation. Longitudinal validation and external cohort replication are therefore necessary to further evaluate the biological and clinical stability of the identified phenotypes. Overall, the present findings support systems phenotyping as a useful approach for revealing endocrine–metabolic organization patterns beyond isolated insulin resistance-associated biochemical abnormalities.

Several directions for future research emerge from the present findings. First, prospective longitudinal validation studies are needed to assess the temporal stability and clinical predictive value of the identified endocrine–metabolic phenotypes, particularly for the incidence of T2D, cardiovascular outcomes, and the progression of hepatic steatosis. Second, external replication of the phenotypic structure across geographically and ethnically distinct cohorts would help establish the generalizability of the identified endocrine–metabolic organization, including the partial-correlation architecture between the metabolic and thyroid blocks, beyond the present single-center setting. Third, integration of additional biological dimensions could further refine phenotype characterization. The relevant additions include chronic low-grade inflammatory biomarkers, adipokines, body composition measures, and circulating metabolomic or transcriptomic signatures, which may clarify the mechanisms linking insulin resistance, hepatic remodeling, and thyroid autoimmunity. Finally, the clinical utility of the systems phenotyping approach should be evaluated as a decision-support tool for endocrine–metabolic risk stratification and personalized monitoring in patients with thyroid dysfunction, within prospective, multicentric study designs. If validated over time, the identified phenotypic structure could inform individualized risk stratification and follow-up for patients with coexisting thyroid dysfunction and metabolic burden. In particular, the distinction between hepatic-dominant and insulin-resistance-dominant patterns could be incorporated into electronic health record decision-support tools, facilitating adoption in routine endocrinology practice.

## 5. Conclusions

Three clinically interpretable phenotypic partitions of a data-driven metabolic continuum were identified in patients with thyroid dysfunction. TyG values aligned with the phenotypic ordering and emerged as an accessible single-number descriptor of a coordinated insulin-resistance–hepatic–adiposity pattern. Notably, the identification of an intermediate hepatic-dominant phenotype suggests that hepatic dysregulation may represent a distinct stage within the broader metabolic continuum, rather than merely a consequence of advanced insulin resistance. Partial-correlation network analysis showed that metabolic and thyroid variables form two nearly orthogonal dimensions of the endocrine–metabolic system, indicating that thyroid-related variation acts as a modulatory layer parallel to the principal metabolic structure. Together, these findings support the utility of systems phenotyping for revealing endocrine–metabolic organization beyond isolated biochemical abnormalities and position TyG as an accessible descriptor of coordinated metabolic remodeling.

## Figures and Tables

**Table 1:** Baseline characteristics of the study cohort according to TyG quartiles.

Variable	Overall	Q1	Q2	Q3	Q4	*p*-Value
Glucose	91.00 (83.00–105.50)	84.00 (77.00–91.00)	90.00 (80.00–93.00)	93.50 (85.75–102.00)	114.00 (96.00–134.00)	<0.001
Triglycerides	112.00 (85.00–142.50)	69.00 (58.00–79.00)	104.00 (91.00–112.00)	130.50 (112.00–139.50)	173.00 (143.00–204.00)	<0.001
TyG	8.55 (8.21–8.86)	7.99 (7.86–8.11)	8.39 (8.28–8.52)	8.70 (8.61–8.79)	9.16 (8.99–9.40)	<0.001
HDL	52.00 (44.00–62.55)	61.50 (51.00–71.00)	52.00 (48.00–62.00)	51.90 (41.48–61.12)	46.00 (38.00–53.00)	<0.001
LDL	112.00 (86.00–140.00)	99.00 (84.00–127.00)	112.00 (88.00–130.00)	124.00 (95.75–149.50)	113.00 (84.00–142.00)	0.005
BMI	28.88 (25.20–32.71)	25.32 (22.68–28.88)	28.88 (25.40–32.89)	29.15 (26.98–33.10)	31.00 (28.52–35.70)	<0.001
ALT	22.00 (17.00–28.00)	22.00 (15.00–28.00)	22.00 (17.00–27.00)	23.50 (18.00–29.00)	24.00 (18.00–31.00)	0.036
AST	22.00 (19.00–27.00)	22.00 (19.00–28.00)	21.00 (18.00–24.00)	23.00 (20.00–28.00)	22.00 (19.00–27.00)	0.018
Uric acid	5.00 (4.10–6.20)	4.20 (3.65–5.40)	4.75 (4.07–5.83)	5.30 (4.30–6.55)	5.55 (4.60–6.78)	<0.001
TSH	1.39 (0.64–2.53)	1.24 (0.64–2.20)	1.24 (0.60–2.47)	1.54 (0.70–2.91)	1.42 (0.67–2.85)	0.534
FT4	14.61 (12.79–16.93)	14.73 (13.44–16.87)	14.73 (13.71–16.30)	14.72 (12.53–16.91)	14.27 (12.20–17.26)	0.647
antiTPO	39.65 (28.00–228.78)	32.80 (28.00–77.60)	43.80 (28.00–394.70)	40.00 (28.00–104.60)	39.65 (28.00–429.32)	0.328
antiTG	1.20 (0.70–4.05)	1.00 (0.80–3.50)	1.00 (0.70–1.77)	1.40 (0.62–11.70)	1.60 (0.70–6.90)	0.204

Notes: Continuous variables are summarized as median (IQR) computed from the working dataset (N = 387). *p*-values were obtained with the Kruskal–Wallis H test across TyG quartiles. Abbreviations: TyG, triglyceride–glucose index; HDL, high-density lipoprotein cholesterol; LDL, low-density lipoprotein cholesterol; BMI, body mass index; ALT, alanine aminotransferase; AST, aspartate aminotransferase; TSH, thyroid-stimulating hormone; FT4, free thyroxine; antiTPO, anti-thyroid peroxidase antibodies; antiTG, anti-thyroglobulin antibodies.

**Table 2:** Clinical and Endocrine-Metabolic Characteristics Across the Data-Driven Phenotypes.

Variable	Metabolically Preserved (*n* = 185)	Intermediate Hepatic-Dominant (*n* = 57)	Severe Insulin-Resistance Dominant (*n* = 145)	*p*-Value
Age	55.0 (47.0–66.0)	54.0 (44.0–63.0)	59.0 (50.0–66.0)	0.143
Female sex	155/184 (84.2%)	35/57 (61.4%)	116/145 (80.0%)	0.001
Fasting glucose (mg/dL)	87.0 (80.0–95.0)	98.0 (85.0–131.0)	96.0 (89.0–112.8)	<0.001
Triglycerides (mg/dL)	88.0 (69.0–111.0)	120.0 (99.0–161.0)	141.0 (118.0–177.0)	<0.001
HDL cholesterol (mg/dL)	61.0 (52.0–70.0)	47.0 (38.9–55.0)	45.0 (39.0–52.0)	<0.001
LDL cholesterol (mg/dL)	112.0 (89.0–136.0)	103.0 (75.0–136.0)	117.0 (86.0–143.0)	0.098
BMI (kg/m^2^)	26.70 (23.53–28.88)	28.88 (25.42–33.60)	32.11 (28.88–36.99)	<0.001
ALT (U/L)	20.0 (15.0–25.0)	47.0 (38.0–62.0)	21.0 (17.0–26.0)	<0.001
AST (U/L)	22.0 (18.0–25.0)	38.0 (31.0–51.0)	21.0 (18.0–23.0)	<0.001
Uric acid (mg/dL)	4.25 (3.60–5.40)	5.30 (4.15–6.45)	5.60 (4.67–6.65)	<0.001
TyG index	8.24 (7.99–8.53)	8.74 (8.44–9.04)	8.87 (8.65–9.16)	<0.001
TSH (mIU/L)	1.30 (0.66–2.37)	1.42 (0.76–3.71)	1.46 (0.53–2.42)	0.547
FT4 (pmol/L)	14.92 (13.73–16.59)	14.30 (12.12–16.76)	14.27 (12.53–16.97)	0.183
antiTPO (IU/mL)	40.4 (28.0–242.8)	34.5 (28.0–57.3)	40.3 (28.0–417.0)	0.453
antiTG (IU/mL)	1.20 (0.70–4.00)	1.10 (0.60–2.55)	1.15 (0.60–4.20)	0.727
Obesity (BMI ≥ 30)	27/185 (14.6%)	25/57 (43.9%)	98/145 (67.6%)	<0.001
Hepatic steatosis	23/185 (12.4%)	20/57 (35.1%)	45/145 (31.0%)	<0.001
Hypothyroidism (TSH > 4.5)	19/170 (11.2%)	13/53 (24.5%)	13/141 (9.2%)	0.013
antiTPO positivity	69/116 (59.5%)	18/35 (51.4%)	62/103 (60.2%)	0.648
antiTG positivity	21/85 (24.7%)	4/19 (21.1%)	18/68 (26.5%)	0.882
Autoimmune thyroiditis proxy	73/122 (59.8%)	19/36 (52.8%)	64/104 (61.5%)	0.736
Hyperuricemia (> 6)	14/102 (13.7%)	17/47 (36.2%)	42/108 (38.9%)	<0.001

Notes: Continuous variables are summarized as median (IQR). Categorical variables are presented as counts (number of measurements) and percentages (% of measurements), so denominators reflect variable-specific availability. *p*-values were obtained with the Kruskal–Wallis H test for continuous variables and the chi-square test for categorical variables. Obesity was defined as BMI ≥ 30 kg/m^2^; hypothyroidism as TSH > 4.5 mIU/L; antiTPO positivity as antiTPO > 34 IU/mL; antiTG positivity as antiTG > 4 IU/mL; hyperuricemia as uric acid > 6 mg/dL; the autoimmune thyroiditis proxy is defined as antiTPO and/or anti-TG positivity in patients with at least one autoantibody measurement. Continuous thyroid autoantibody values (anti-TPO, anti-TG) were available for only a subset of patients; the corresponding measured denominators are shown in the antiTPO and antiTG positivity rows below. *p*-values for the continuous antibody variables refer to the measured subset and should be interpreted accordingly.

**Table 3:** Thyroid-associated sub-phenotypes within the data-driven phenotypes.

Main Phenotype	Sub-Phenotype	TSH	*p*-Value	FT4	*p*-Value	antiTPO	*p-*Value	antiTG	*p*-Value
Metabolically Preserved	Thyroid-autoimmune low	1.18 (0.61–2.05)	<0.001	14.88 (13.42–16.61)	0.412	28.00 (28.00–41.30)	<0.001	1.20 (0.80–1.30)	<0.001
Metabolically Preserved	Thyroid-autoimmune dominant	2.42 (1.26–4.96)	<0.001	14.63 (12.74–16.09)	0.412	1180.00 (512.40–1300.00)	<0.001	2.60 (1.30–18.50)	<0.001
Intermediate Hepatic-Dominant	Thyroid-autoimmune low	1.36 (0.72–2.94)	0.083	14.11 (12.48–16.08)	0.218	31.60 (28.00–45.90)	<0.001	1.20 (0.80–1.30)	0.009
Intermediate Hepatic-Dominant	Thyroid-autoimmune dominant	2.01 (0.94–4.41)	0.083	13.74 (11.96–15.82)	0.218	1300.00 (688.20–1300.00)	<0.001	12.40 (2.40–56.30)	<0.001
Severe Insulin-Resistance	Thyroid-autoimmune low	1.27 (0.59–2.18)	0.012	14.37 (12.88–16.92)	0.337	28.00 (28.00–38.70)	<0.001	1.30 (0.80–1.30)	<0.001
Severe Insulin-Resistance	Thyroid-autoimmune dominant	2.76 (1.44–5.22)	0.012	13.91 (12.31–16.10)	0.337	944.00 (401.20–1300.00)	<0.001	8.10 (1.70–44.20)	<0.001

Notes: Values are presented as median and interquartile range (IQR). Sub-phenotype assignment was based on the autoantibody status of patients with available anti-TPO or anti-TG measurements (overall available: anti-TPO 254/387; anti-TG 172/387). Patients without measurements were excluded from this descriptive analysis. Within each phenotype, sub-phenotypes were compared using the Mann–Whitney U test separately for TSH, FT4, anti-TPO, and anti-TG. Because of substantial missingness in autoantibody measurements and because sub-phenotypes were defined and characterized using the same thyroid variables, these findings should be interpreted with caution and as descriptive rather than as independent validation. TyG, triglyceride-glucose index; TSH, thyroid-stimulating hormone; FT4, free thyroxine; antiTPO, anti-thyroid peroxidase antibodies; antiTG, anti-thyroglobulin antibodies.

## Data Availability

The data presented in this study are available on request from the corresponding author. Access is restricted because the dataset contains sensitive patient health information and is subject to the ethical approval conditions granted by the Institutional Ethics Committee, which limits open dissemination to protect participant confidentiality. Requests for data access may be directed to the corresponding authors.

## Abbreviations

The following abbreviations are used in this manuscript:

ALT	alanine aminotransferase
antiTG	anti-thyroglobulin antibodies
antiTPO	anti-thyroid peroxidase antibodies
ARI	Adjusted Rand Index
AST	aspartate aminotransferase
BIC	Bayesian information criterion
BMI	body mass index
FT4	free thyroxine
GGM	Gaussian graphical model
GMM	Gaussian mixture model
HDL	high-density lipoprotein cholesterol
IHD	intermediate hepatic-dominant
IQR	interquartile range
IR	insulin resistance
LDL	low-density lipoprotein cholesterol
LPA	latent profile analysis
MP	metabolically preserved phenotype
MetS	metabolic syndrome
NAFLD	non-alcoholic fatty liver disease
PCA	principal component analysis
SIRD	severe insulin-resistance-dominant
STROBE	Strengthening the Reporting of Observational Studies in Epidemiology
T2DM	type 2 diabetes mellitus
TG	triglycerides
TSH	thyroid-stimulating hormone
TyG	triglyceride–glucose index
